# Role of Folic acid as adjuvant treatment in Schizophrenia: A randomized controlled trial

**DOI:** 10.1192/j.eurpsy.2023.1321

**Published:** 2023-07-19

**Authors:** P. Saxena, S. Kumar, K. Vaibhav

**Affiliations:** 1 Psychiatry, Rajashri Dashrath Autonomous State Medical College, Ayodhya; 2 Psychiatry, Hind Institute of Medical Sciences, Lucknow, India

## Abstract

**Introduction:**

Schizophrenia is a chronic psychiatric illness with symptoms in positive, negative and cognitive domain.The interplay of dietary folic acid intake with common genetic variants that influence folate metabolism, has potential implications for Schizophrenia pathogenesis and treatment.Therefore, it’s deficiency has been identified as a risk factor for Schizophrenia through epidemiologic, biochemical and gene association studies.

**Objectives:**

1-To assess the efficacy of folic acid supplementation on severity of symptoms and overall functional status of patients

2-To assess the correlation of serum folate levels with symptom severity and overall functional status of patients

**Methods:**

A randomized control trial study was carried out in the inpatient department of a psychiatric tertiary care centre on 40 participants (29 males and 11 females)who were between the ages of 18 – 55 years,met diagnostic criteria for Schizophrenia (ICD 10) and had at least 2 years of illness duration while those with a co-morbid psychiatric illness, medical illness and substance abuse were excluded. The participants were then randomly allocated into two groups (**experimental Group A** which received 5mg folic/day along with anti psychotic drugs and **control Group B** which received only anti psychotic drugs) and followed up for 3 months. Blood sample for measuring serum folate level was obtained from the experimental group at the beginning and at the end of the study period. Scales applied were Positive and Negative Syndrome Scale(PANSS) for symptom severity and Global Assessment of Functioning scale(GAF) for overall functional status.

**Results:**

A significant difference (p value< 0.05) was observed in PANSS scores at the end of the study between experimental group and control group( **table 1**) and also in GAF scores between both the groups after 3 months(**table 2**). At the end of the study period,a strong negative correlation(r= -0.9) was found between serum folate level and total PANSS score in the experimental group (**figure 1**) while the correlation between GAF score and serum folate level was strongly positive (r= 0.8) (**figure 2**).

**Table 1:** 

**PANSS (3 Month)**	**Group A(n=20)**	**Group B (n=20)**	**P value**
**Positive**	16.8±2.80	22.9±3.37	0.036
**Negative**	14.3±3.32	15.1±2.61	0.18
**General**	17.95±2.52	21.85±3.18	0.0001
**Total**	45.95±3.41	58±3.49	0.00249

**Table 2:** 

**GAF**	**Group A(n=20)**	**Group B (n=20)**	**P value**
**0 MONTH**	23.25±3.43	22.7±2.90	0.3
**3 MONTH**	65.75±4.22	44.9±7.09	0.0256

**Image:**

**Figure:**
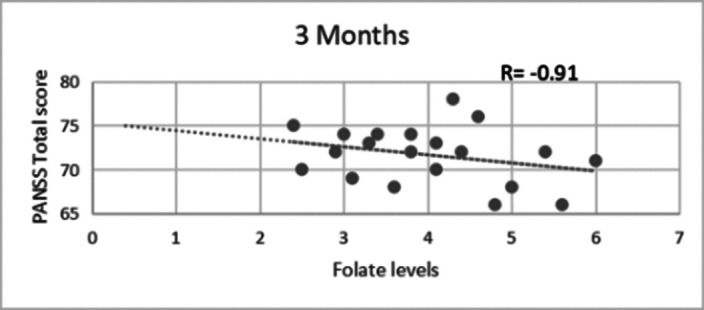

**Image 2:**

**Figure:**
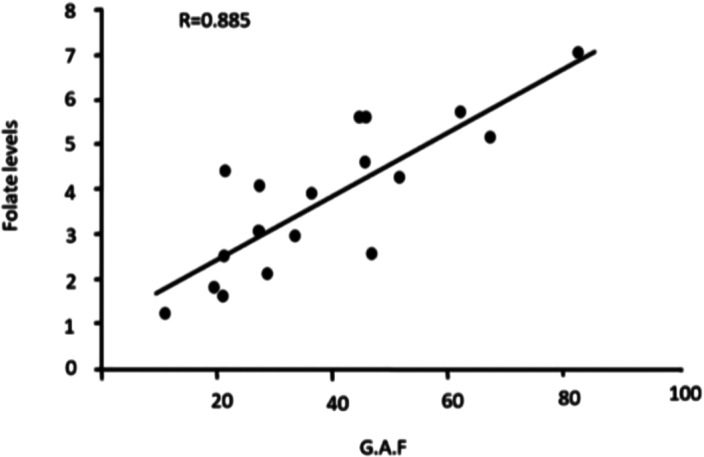

**Conclusions:**

Our study is among the few to use a randomized controlled study design for assessing the effect of folic acid supplementation on severity of symptoms and global functioning in Schizophrenia,strongly suggesting the use of folic acid as an adjuvant treatment for Schizophrenia.

**Disclosure of Interest:**

None Declared

